# Unlocking the Potential of Nutraceuticals in Cancer Chemotherapy: A Comprehensive Review

**DOI:** 10.7759/cureus.89328

**Published:** 2025-08-04

**Authors:** Pravesh Aggarwal, Gitashree Dutta, Rekha Shaurya, Vinoth Rajendran, Prasanna Thirunavukkarasu, Jaykaran Charan, Tarun Kumar

**Affiliations:** 1 Pharmacology, All India Institute of Medical Sciences, Jodhpur, Jodhpur, IND; 2 Community Medicine, Government Medical College, Pali, Pali, IND; 3 Pharmacology, Dr. Sampurnanand Medical College, Jodhpur, IND; 4 Community Medicine & Family Medicine, All India Institute of Medical Sciences, Gorakhpur, Gorakhpur, IND; 5 Community Medicine and Family Medicine, All India Institute of Medical Sciences, Jodhpur, Jodhpur, IND

**Keywords:** antioxidants, cancer chemotherapy, chemotherapy side effects, curcumin, gut microbiota, integrative oncology, nutraceuticals

## Abstract

Cancer remains a leading global cause of morbidity and mortality. While conventional therapies such as chemotherapy and radiation are essential, growing evidence supports the adjunctive use of nutraceuticals-bioactive compounds from natural food sources-with anticancer potential. To synthesize current evidence on the mechanisms, therapeutic roles, and clinical challenges of nutraceuticals in cancer chemotherapy.

A narrative review was conducted by searching literature from 2015 to 2024 across PubMed, Scopus, and Web of Science. Studies were included based on relevance to cancer patients, nutraceutical interventions, and reported therapeutic or supportive outcomes. No formal meta-analysis was performed; findings were thematically grouped and summarized. Nutraceuticals such as curcumin, resveratrol, flavonoids, and vitamins D and E exert antioxidant, anti-inflammatory, pro-apoptotic, and immune-modulatory effects. Many demonstrate synergy with chemotherapy, enhancing efficacy and reducing toxicity. Probiotics and omega-3 fatty acids, in particular, show promise in alleviating chemotherapy-induced side effects. However, clinical utility is limited by inconsistent trial results, variable dosing, low bioavailability, and lack of regulatory oversight. Some supplements may interfere with standard therapies or pose safety concerns if used indiscriminately.

Nutraceuticals offer promising adjunctive benefits in cancer care, but their integration requires cautious, evidence-based application. Standardized formulations, better clinical trial designs, and regulatory clarity are needed to ensure their safe and effective use in oncology.

## Introduction and background

Introduction

Cancer, characterized by abnormal and uncontrolled cell growth, remains one of the leading causes of mortality worldwide [1]. Projections indicate that cancer-related mortality could reach 18 million by 2050, underscoring the urgent need for complementary strategies to improve treatment outcomes and reduce therapy-related toxicity [1]. One such strategy is the use of nutraceuticals, dietary supplements that deliver concentrated biologically active components of food in non-food matrices to support health and disease prevention [2].

The etiology of cancer is multifactorial, involving both genetic and somatic mutations, influenced by environmental and lifestyle factors [3,4]. Contributing factors include infections, chronic inflammation, radiation exposure, occupational stress, poor dietary patterns, and toxins [5]. Diets rich in processed, pickled, or salted meats are associated with an increased risk of gastric cancer, while high-fat, low-fiber diets are linked to cancers of the breast, pancreas, endometrium, ovary, colon, and prostate [6,7]. Additionally, overeating is implicated in approximately 30% of cancer incidences [5].

Standard cancer therapies such as surgery, chemotherapy, and radiation aim to reduce tumor burden but often lack specificity, harming healthy tissues and causing adverse effects such as immunosuppression, fatigue, and organ toxicity. Over time, resistance to these treatments may also develop, limiting their long-term efficacy [8,9]. While advanced modalities like immunotherapy and gene therapy are emerging, their high cost and limited accessibility remain significant barriers [10].

In this context, nutraceuticals are being explored as adjunctive agents due to their potential to enhance therapeutic outcomes and reduce side effects. These compounds modulate various pathways involved in carcinogenesis, including cell proliferation, apoptosis (cell death), angiogenesis, and inflammation [11]. Some exhibit antioxidant effects, reduce DNA damage, modulate epigenetic mechanisms, restore the activity of tumor suppressor genes, and improve immune surveillance. Others may influence gut microbiota, which is increasingly recognized for its role in systemic inflammation and immune modulation.

Sourced largely from nature, nutraceuticals offer health benefits beyond basic nutrition [12]. They include nutrients (vitamins, minerals, amino acids, and fatty acids), herbal products (garlic, ginger, goldenseal, and melissa), dietary supplements (black cohosh, ginkgo biloba, and glucosamine), probiotics (*Bifidobacterium* and *Lactobacillus*), and prebiotics (galacto-oligosaccharide and inulin) [13]. Common examples include lycopene in tomatoes, omega-3 fatty acids in salmon, and saponins in soy. Fortified foods, such as calcium-enriched orange juice and folic acid-enriched flour, also serve as sources of nutraceuticals [13].

Despite promising preclinical data, the clinical translation of nutraceuticals in oncology remains limited due to variability in trial design, dosage, formulation, and regulatory oversight. Robust, standardized clinical trials are essential to validate their efficacy, safety, and role in integrated cancer care.

Rationale for Review: Given the growing interest in dietary interventions for cancer, this review aims to evaluate the mechanistic basis, clinical impact, and translational potential of nutraceuticals in cancer chemotherapy.

## Review

Methods

This narrative review was developed in accordance with established guidelines for narrative synthesis. A comprehensive literature search was conducted using PubMed, Cochrane, Scopus, and Google Scholar, employing specific search terms such as “nutraceuticals”, “dietary supplements”, “functional foods”, “phytochemicals”, “natural compounds”, and “cancer”.

Eligible studies were limited to full-text, English-language publications from the past five years. Inclusion criteria encompassed clinical trials, randomized controlled trials (RCTs), observational studies, meta-analyses, and review articles. Editorials, correspondences, and case reports were excluded.

The findings were synthesized narratively, emphasizing the mechanistic roles of nutraceuticals and their relevance to cancer chemotherapy. Data extraction was performed qualitatively, focusing on intervention types and reported clinical or mechanistic outcomes. Due to heterogeneity in study designs, populations, interventions, and endpoints, no formal quality appraisal or meta-analysis was conducted.

As this review involved only secondary analysis of published data, ethical approval and protocol registration were not required.

Mechanisms of action of nutraceuticals in cancer

Nutraceuticals exert anticancer effects by various mechanisms.

Antioxidant and Chemoprotective Effects

One of the key mechanisms by which nutraceuticals act is through antioxidant activity. Oxidative stress results from an imbalance between reactive oxygen species (ROS) and the body’s antioxidant defense [14]. This can lead to DNA damage, mutations, and aberrant cell signaling, all of which contribute to cancer initiation and progression [15]. Nutraceuticals with antioxidant properties help scavenge free radicals, enhance cellular defense systems, and protect normal tissues from the cytotoxic effects of chemotherapy, thereby improving its therapeutic index [16].

For instance, hydroxylated derivatives of resveratrol, such as dihydroxystilbenes and tetrahydroxystilbene, offer significant oxidative protection [17]. Tinospora cordifolia (Guduchi/Giloy), widely used in traditional medicine, has shown both antioxidant and immunomodulatory effects, with preclinical and clinical data suggesting it can strengthen endogenous defenses, promote cancer cell apoptosis, and improve quality of life during conventional cancer treatment [18]. Quercetin, a dietary flavonoid, scavenges ROS, enhances antioxidant enzyme activity, and protects DNA from oxidative damage [19]. Similarly, curcumin, a well-known polyphenol, suppresses lipid peroxidation and activates the Nrf2 signaling pathway, which regulates genes responsible for cellular antioxidant responses [20]. Sulforaphane, an organosulfur compound found in cruciferous vegetables, induces phase II detoxification enzymes, supporting cellular defenses against oxidative injury [21]. Collectively, these nutraceuticals not only mitigate oxidative damage but may also improve treatment tolerance and therapeutic outcomes in cancer patients.

Direct Anticancer Effects (Apoptosis, Angiogenesis, Proliferation)

Nutraceuticals exhibit direct anticancer activity through multiple mechanisms, primarily by inducing apoptosis, inhibiting angiogenesis, and suppressing cellular proliferation and metastasis. Apoptosis, or programmed cell death, is a key natural defense against malignancy, and many nutraceuticals enhance this process by activating pro-apoptotic proteins while inhibiting cell survival pathways [22]. Angiogenesis, a critical process in tumor growth, and uncontrolled proliferation are also targeted by these compounds through modulation of specific signaling pathways.

Curcumin, a polyphenol from turmeric, disrupts cancer cell formation and tumor development by promoting apoptosis via a p53-dependent mechanism, while also suppressing angiogenesis through nuclear factor-κB (NF-κB) and AP-1 inhibition and downregulating vascular endothelial growth factor (VEGF) and basic fibroblast growth factor. It further reduces IL-8 expression and modulates pathways associated with inflammation, UV-induced mutagenesis, and insulin resistance by targeting insulin-like growth factor-1 (IGF-1) and tumor necrosis factor alpha (TNF-α), particularly relevant to colorectal cancer [17]. Resveratrol derivatives, including methoxylated, hydroxylated, and halogenated forms, exhibit enhanced anticancer and therapeutic properties through diverse mechanisms. Methoxylated derivatives like pterostilbene and trimethoxystilbene inhibit key signaling pathways (e.g., phosphatidylinositol 3-kinase (PI3K)/AKT, signal transducer and activator of transcription (STAT-3), and NF-κB pathways), angiogenesis, and induce apoptosis, showing efficacy against breast, lung, liver, and colon cancers [16]. Hydroxylated derivatives, such as dihydroxystilbenes and tetrahydroxystilbene, provide G1-phase arrest, aiding in the treatment of lung, breast, liver, and leukemia cancers. Halogenated derivatives, including 4’-bromoresveratrol, target enzymes like Sirtuins and transthyretin, offering therapeutic benefits in cancer, cardiovascular, and age-related diseases [17,23,24]. Vitamin D induces anti-angiogenic and apoptotic effects by binding to its receptor and upregulating genes related to DNA damage and growth arrest [16]. Other compounds like Annurca apple Polyphenol Extract (APE) trigger extrinsic, p53-independent apoptosis and inhibit proliferation, showing potential in skin cancer prevention [25]. Anthraquinones, including kwanzoquinone C from Hemerocallis fulva, exhibit broad-spectrum anticancer activity by disrupting the cell cycle, inducing apoptosis, and limiting metastasis [26]. Flavonoids abundant in tomatoes, mulberries, and Amazon grapes, exert anticancer effects through anti-inflammatory, anti-angiogenic, and antioxidant-induced apoptotic mechanisms. It modulates key signaling pathways such as PI3K/Akt JAK/STAT, mitogen-activated protein kinase (MAPK), and NF-κB with particular efficacy in colon, lung, liver, and breast cancers [27]. Sulforaphane (in broccoli) and lycopene (in tomatoes) enhance chemotherapy efficacy by modulating the cell cycle and inducing apoptosis [28,29]. Lycopene reduces cyclin and cyclin-dependent kinase (CDK) activity, limiting uncontrolled cell growth, while sulforaphane suppresses epithelial-mesenchymal transition (EMT), a key step in metastasis [30,31]. Carnosol, a phenolic diterpene found in rosemary and sage, shows potent anticancer effects by downregulating COX-2, p38, JNK, Akt, mammalian target of rapamycin (mTOR), and ERK pathways, promoting apoptosis through decreased Bcl-2, increased Bax, and activation of caspases-3, -8, and -9 [32]. Similarly, quercetin inhibits PI3K/Akt signaling, elevating pro-apoptotic Bax and reducing anti-apoptotic Bcl-2, thereby contributing to apoptosis and growth inhibition in various cancer models [33,34]. Table 1 summarizes the primary nutraceuticals discussed in this section, along with their mechanisms of anticancer action.

**Table 1:** Key Nutraceuticals and Their Mechanisms of Anticancer Action NF-κB: nuclear factor-κB; JAK/STAT: Janus kinases/Signal transducer and activator of transcription; MAPK: mitogen-activated protein kinase; COX-2: cyclooxygenase; PI3K: phosphatidylinositol 3-kinase; HDAC: histone deacetylases; EMT: epidermal to mesenchymal transition; mTOR: mammalian target of rapamycin

Nutraceutical	Source	Primary Mechanisms	Targeted Cancers/Effects
Curcumin	Turmeric	Apoptosis induction (p53), NF-κB/AP-1 inhibition, anti-angiogenic, anti-inflammatory	Breast, colon, lung, colorectal
Resveratrol (incl. derivatives)	Grapes, berries	PI3K/AKT, STAT-3, NF-κB inhibition, G1 arrest, apoptosis induction	Colon, liver, lung, breast, leukemia
Sulforaphane	Broccoli	HDAC inhibition, antioxidant, EMT suppression, phase II detox enzymes	Breast, prostate, multiple cancers (adjunctive)
Vitamin D	Sunlight, fortified foods	Anti-angiogenic, pro-apoptotic, DNA repair gene upregulation	Colorectal
Quercetin	Onions, apples	PI3K/Akt inhibition, ↑Bax, ↓Bcl-2, anti-inflammatory	Lung, leukemia, solid tumors
APE (Annurca Apple Polyphenol Extract)	Annurca apple	p53-independent apoptosis, anti-proliferative	Skin cancer
Anthraquinones	Hemerocallis fulva	Cell cycle disruption, apoptosis induction, anti-metastatic	Broad anticancer activity
Flavonoids	Tomatoes, mulberries, Amazon grapes	Anti-inflammatory, anti-angiogenic, PI3K/Akt, JAK/STAT, MAPK, NF-κB modulation	Colon, lung, liver, breast
Lycopene	Tomatoes	Cyclin/CDK inhibition, cell cycle modulation, pro-apoptotic	Prostate, colon, breast
Carnosol	Rosemary, sage	COX-2, JNK, mTOR, Akt inhibition; caspase activation; apoptosis	Breast, prostate, solid tumors

Epigenetic Modulation

Epigenetic modifications, including DNA methylation, histone alterations, and microRNA expression, play a pivotal role in regulating gene activity without changing the underlying DNA sequence [35]. In cancer, aberrant epigenetic changes often contribute to tumor initiation and progression. Nutraceuticals have shown promise in reversing such modifications, thereby restoring normal gene expression and offering a novel therapeutic avenue [36,37].

For instance, genistein, a soy isoflavone, inhibits DNA methyltransferases, leading to the reactivation of silenced tumor suppressor genes [38]. Sulforaphane, a compound found in cruciferous vegetables, modulates histone deacetylase (HDAC) activity and enhances the expression of pro-apoptotic genes [39,40,41]. A growing body of evidence highlights plant-derived nutraceuticals such as curcumin, resveratrol, sulforaphane, indole-3-carbinol, quercetin, astaxanthin, epigallocatechin-3-gallate, and lycopene for their epigenetic regulatory roles. These compounds influence key targets including HDAC, HAT (histone acetyltransferase), DNA methyltransferases (DNMTs), and non-coding RNAs, collectively contributing to the suppression of oncogenic pathways [39,42]. Such reversible modifications present a low-toxicity strategy for cancer prevention and therapy through the normalization of gene expression.

Immunomodulatory Properties

The immune system is a critical line of defense against tumor development and progression, and enhancing immune function is increasingly recognized as a promising strategy in cancer therapy. Nutraceuticals have emerged as potential agents capable of modulating immune responses to boost antitumor activity while counteracting immune evasion mechanisms employed by cancer cells. Resveratrol enhances the cytotoxicity of CD8⁺ T lymphocytes and natural killer (NK) cells while suppressing the proliferation of regulatory T cells (Tregs), which otherwise dampen antitumor immunity [43,44]. Curcumin contributes to immune regulation within the tumor microenvironment by downregulating pro-inflammatory cytokines such as IL-6 and TNF-α and upregulating anti-inflammatory cytokines like IL-10, thereby creating an immune-favorable environment [45,46]. Vitamin D demonstrates immunomodulatory and chemopreventive effects, particularly in colorectal cancer, by modulating cytokine synthesis, promoting T-cell activation, and reducing inflammation associated with tumor progression [47].

Additionally, other micronutrients like vitamin E have shown potential in enhancing immune surveillance and improving cancer outcomes [48]. Collectively, these nutraceuticals offer promising adjunctive roles in cancer immunotherapy by restoring immune homeostasis and enhancing tumor-specific immune responses.

Modulation of Gut Microbiota

Nutraceuticals play a pivotal role in shaping gut microbiota composition; a key factor increasingly linked to regulating systemic immune responses and therapeutic results in cancer. Anthocyanins, a subclass of flavonoid pigments, promote gut microbiota balance and increase short-chain fatty acid (SCFA) production, particularly butyrate, contributing to systemic anti-inflammatory and anticancer effects. With an established safety profile at doses up to 2.5 mg/kg/day, they represent a promising adjunct in both cancer prevention and treatment strategies [49]. Nutraceuticals such as dietary fibers, polyphenols, and probiotics support the growth of beneficial microbes like Lactobacillus and Bifidobacterium, which produce SCFAs with known anti-carcinogenic properties [50,51]. These interventions not only suppress pathogenic bacteria but also regulate estrogen metabolism, immune responses, and inflammatory pathways, helping to restore the balance between cell proliferation and apoptosis, particularly in colorectal cancer. Thus, targeted modulation of gut microbial health using nutraceuticals offers a novel, low-toxicity approach for cancer prevention and as an adjunct to conventional therapies [52].

Clinical utility and evidence

Clinical Trials and Meta-Analyses

Although numerous in vitro and in vivo studies have demonstrated the anticancer potential of nutraceuticals, their translation into clinical practice remains limited due to challenges related to study design, bioavailability, and regulatory inconsistencies. Regardless, there have been several clinical trials that have explored and provided data on the use of nutraceuticals in cancer prevention and treatment. Curcumin, among the most extensively studied nutraceuticals, was shown to be safe and well-tolerated at doses up to 8 g/day in a phase I trial [53]. Other studies have examined curcumin in combination with chemotherapy, reporting improved treatment tolerability and reductions in inflammatory biomarkers such as NF-κB and COX-2 [54,55]. A recent randomized clinical trial demonstrated that weekly oral vitamin D supplementation during neoadjuvant systemic therapy significantly improved pathologic complete response rates in breast cancer patients, particularly in those with hormone receptor-negative tumors and high Ki-67 expression [56]. Further evidence supports vitamin D supplementation as being associated with better overall and recurrence-free survival in early-stage adenocarcinoma patients with low baseline levels [57]. In colorectal cancer, vitamin E has been shown to enhance NK cell activity, potentially improving immune-mediated cancer surveillance and complementing chemotherapy, though more research is needed to define optimal dosing and mechanisms [48]. A double-blind randomized trial found that a rosemary-derived bioactive formulation reduced systemic inflammation and modulated immune function in lung cancer patients, supporting its use as a complementary intervention [58]. Similarly, a randomized trial in head and neck cancer patients receiving home enteral nutrition revealed that resveratrol supplementation improved antioxidant enzyme activity and cellular health, indicating potential for supportive oncologic care [59]. However, a phase II randomized trial combining curcumin and anthocyanins in patients with adenomatous polyps did not yield significant changes in inflammatory or metabolic biomarkers, though it revealed complex biomarker interactions associated with dysplasia and colorectal cancer risk [60].

Synergistic Effects with Chemotherapy

A compelling feature of nutraceuticals is their ability to act synergistically with standard chemotherapy regimens. This can help patients who are resistant to common chemotherapy drugs. By targeting complementary molecular pathways, nutraceuticals can amplify the cytotoxic effects of chemotherapy while minimizing its adverse effects. For instance, curcumin has been shown to enhance the efficacy of drugs such as 5-fluorouracil, doxorubicin, and cisplatin by sensitizing cancer cells to treatment [61]. This effect is partly mediated through the inhibition of pro-survival signaling pathways and the downregulation of multidrug resistance proteins [62]. Additionally, high levels of folate in the culture medium have been found to increase the sensitivity of adenocarcinoma cells to cisplatin, suggesting a synergistic interaction that could be exploited therapeutically [63].

Management of Chemotherapy Side Effects

Nutraceuticals are also very helpful in controlling the side effects of chemotherapy, such as nausea, exhaustion, and immunosuppression. Incorporating omega-3 fatty acids into chemotherapy regimens has been linked to improved clinical outcomes and reduced treatment-related toxicity [64]. Similarly, ginger supplementation has shown significant efficacy in alleviating chemotherapy-induced nausea, acute vomiting, and fatigue [65]. Furthermore, a meta-analysis of eight RCTs involving 633 colorectal cancer patients demonstrated that probiotic supplementation significantly reduced the incidence of chemoradiotherapy-induced diarrhea and improved symptoms such as pain, dyspnea, and insomnia [66]. These results highlight the preventive function of probiotics in preserving gut health and improving resistance to therapy. Collectively, such supportive effects highlight the potential of nutraceuticals as valuable adjuncts in cancer therapy, improving patient quality of life and comfort during aggressive treatments.

Nutrigenomics and Personalized Nutrition

Nutrigenomics is an emerging field that explores how individual genetic variations influence the body’s response to dietary components. This approach holds significant promise for personalizing nutraceutical interventions in cancer therapy. For example, individuals with polymorphisms in the methylenetetrahydrofolate reductase (MTHFR) gene - critical for folate metabolism - may derive greater benefit from specific forms or doses of folate supplementation [67]. Similarly, single-nucleotide polymorphisms (SNPs) in the vitamin D receptor (VDR) gene can affect the efficacy of vitamin D therapy in colorectal cancer patients [68].

By combining genetic testing with tailored dietary recommendations, personalized nutrition aims to improve therapeutic outcomes, reduce adverse effects, and optimize the integration of nutraceuticals into cancer care. Achieving this precision approach will require close collaboration among oncologists, nutritionists, and molecular biologists to translate nutrigenomic insights into clinical practice.

Limitations and challenges

While nutraceuticals have demonstrated significant potential in preclinical models and early-phase clinical studies, several limitations and challenges hinder their integration into mainstream cancer therapy.

Inconsistent Clinical Evidence

Despite promising preclinical outcomes, the translation of nutraceutical efficacy into clinical trials remains inconsistent. For example, nano-curcumin, despite its enhanced bioavailability, failed to prevent cisplatin-induced nephrotoxicity compared to placebo in a controlled trial (Table 2) [69].

**Table 2:** Clinical Evidence on Nutraceuticals in Cancer Patients NF-κB: nuclear factor-κB; COX-2: cyclooxygenase; HR: hormone receptor; NK: natural killer

Nutraceutical	Study Design	Findings	Cancer Type	Reference
Curcumin	Phase I trial	Safe up to 8 g/day	General	[56]
Curcumin	Clinical study	Improved chemo tolerability; reduced NF-κB and COX-2	Various	[57,58]
Vitamin D	RCT	Improved pathologic complete response in high Ki-67, HR-negative breast cancer	Breast	[59]
Vitamin D	Clinical study	Improved overall and recurrence-free survival in low baseline levels	Colorectal	[26]
Vitamin E	Clinical study	Enhanced NK cell activity; immunostimulatory potential	Colorectal	[51]
Rosemary Extract	Double-blind RCT	Reduced systemic inflammation; improved immune modulation	Lung	[60]
Resveratrol	RCT with enteral nutrition	Improved antioxidant enzyme activity, cellular health	Head and neck	[61]
Curcumin + Anthocyanin	Phase II RCT	No significant biomarker change; complex interactions found	Colorectal (adenomatous polyps)	[62]

Similarly, while vitamin supplementation can improve quality of life and treatment response in deficient individuals, excessive intake in patients with normal baseline levels may act as pro-oxidants and even promote tumor growth. This underscores the importance of maintaining nutrient intake within physiological ranges, ideally through balanced dietary patterns like the Mediterranean diet [70].

Moreover, a systematic review of dietary supplements in breast cancer care revealed mixed results-some supplements improved fatigue and immune responses, while others lacked consistent clinical efficacy or posed risks of adverse interactions with standard therapies [71]. These inconsistencies highlight the need for cautious, individualized application of nutraceuticals in oncology, supported by professional guidance and rigorous clinical evaluation.

Safety, Toxicity, and Drug Interactions

Certain nutraceuticals, when consumed in high doses or without supervision, may lead to adverse effects such as hormonal imbalances, cytotoxicity, or pro-oxidant activity. Overconsumption has been linked to hepatotoxicity, gastrointestinal discomfort, and immune suppression. Furthermore, nutraceuticals can interfere with cytochrome P450 enzymes and other metabolic pathways, altering the pharmacokinetics of chemotherapeutic agents, immunotherapies, or hormone therapies. These interactions may result in either diminished treatment efficacy or heightened toxicity, necessitating careful assessment of potential risks when co-administering nutraceuticals with conventional cancer therapies [72].

Bioavailability and Delivery Barriers

One of the critical challenges in translating the anticancer potential of nutraceuticals into clinical benefit lies in the discrepancy between the concentrations effective in vitro and those achievable in human plasma after oral administration. While many compounds demonstrate potent anticancer activity at micromolar concentrations in cell-based studies, they typically reach only nanomolar levels systemically in humans. This pharmacokinetic gap is further compounded by extensive first-pass metabolism in the liver and gastrointestinal tract, which generates various bioactive metabolites that are often neglected in studies focusing solely on the parent compound.

Nutraceuticals such as curcumin, quercetin, and resveratrol are particularly limited by poor aqueous solubility, low gastrointestinal absorption, and rapid systemic clearance, all of which contribute to subtherapeutic plasma levels and diminished clinical efficacy [73]. Addressing these limitations requires the implementation of innovative strategies to enhance bioavailability. These include the use of nanoformulations, liposomal delivery systems, adjuvants like piperine, and specific preparation or consumption methods [74-77], as outlined in Table 3.

**Table 3:** Practical Strategies to Enhance Bioavailability and Efficacy of Nutraceuticals Through Preparation and Consumption Methods. ALA: alpha-linolenic acid

Nutraceutical	Food Source	Optimization Method	Rationale
Sulforaphane	Broccoli, Brussels sprouts	Consume raw or lightly steamed; chop or chew before eating; add mustard powder after cooking	Light steaming preserves myrosinase enzyme; mustard powder restores enzymatic activity post-cooking [74]
Curcumin	Turmeric	Take with black pepper and healthy fats	Piperine in black pepper enhances bioavailability by inhibiting hepatic metabolism [75]
Resveratrol	Grapes, red wine, peanuts	Consume with meals rich in fat	Fat enhances absorption and stability during digestion [76]
Omega-3 Fatty Acids	Fatty fish (salmon, mackerel), flaxseeds	Prefer baked or steamed fish over frying; grind flaxseeds before use	Heat-sensitive; frying degrades omega-3s; ground flax improves ALA bioavailability [77]

To further address these barriers, advanced delivery systems such as nanoparticles, liposomes, solid lipid carriers, and micelles have been developed to improve bioavailability and target-site accumulation. For example, nano-curcumin encapsulated in poly lactic-co-glycolic acid (PLGA) enhances its cellular uptake, plasma half-life, and antitumor effects compared to unformulated curcumin [78]. Similarly, lipid-based formulations of resveratrol and quercetin demonstrate improved intestinal absorption and bioactivity in preclinical cancer models [79]. These nanocarrier-based systems offer improved pharmacokinetics, reduced off-target toxicity, and controlled release, thus bridging the gap between laboratory potential and clinical translation.

Regulatory and Quality Control Issues

The lack of stringent regulatory oversight remains a major barrier to the clinical integration of nutraceuticals. In the USA, the FDA regulates them as dietary supplements under the DSHEA (1994), exempting them from pre-market approval [80]. In the EU, regulation falls under the European Food Safety Authority (EFSA) guidelines, not the European Medicines Agency (EMA) [81]. These frameworks lead to inconsistent quality, limited safety validation, and product variability. Studies have reported issues such as inconsistent bioactive content, contamination, and misleading health claims. Despite recent improvements in post-market surveillance and Good Manufacturing Practices (GMP) enforcement, the absence of standardized regulation and efficacy data continues to hinder clinical adoption.

Future directions

Nutraceuticals hold considerable promise as adjuncts in cancer therapy, but their successful integration into mainstream clinical practice requires a strategic, multi-faceted approach. A foundational step involves establishing globally harmonized regulatory frameworks, enforcing GMP, and mandating transparent labeling. These regulatory measures are essential to ensure product safety, consistency, and reproducibility - factors that can enhance both clinician trust and patient outcomes in oncology settings.

Personalized nutrition, guided by genomic and metabolic profiling, offers the potential to tailor nutraceutical use based on individual patient characteristics. AI and computational biology are accelerating discovery by predicting compound efficacy and mapping interactions with cancer pathways.

To overcome poor bioavailability, advanced delivery systems - such as nanoparticles, liposomes, and hydrogels - are being developed to enhance absorption, prolong circulation, and minimize toxicity.

Collectively, these innovations mark a paradigm shift in how nutraceuticals may be optimized, personalized, and deployed as adjuncts in cancer therapy. Future large-scale, multicenter clinical trials will be critical in validating these approaches and translating them into routine oncologic practice.

## Conclusions

Nutraceuticals exert diverse mechanisms, including antioxidant, anti-inflammatory, and pro-apoptotic effects that can complement standard cancer therapies. They improve treatment efficacy and minimize the side effects of standard chemotherapy drugs. They improve the overall health of a cancer patient and present a promising supplement to traditional cancer therapy.

Their incorporation in oncology may be vital; however, clinicians should stay vigilant while giving nutraceuticals to prevent an untoward reaction. Patient factors such as cancer type, stage, metabolic state, and current treatments of the cancer must be taken into consideration. More robust clinical trials are needed to prove their efficacy and safety profile.
